# Morphological and chemical stability of silicon nanostructures and their molecular overlayers under physiological conditions: towards long-term implantable nanoelectronic biosensors

**DOI:** 10.1186/1477-3155-12-7

**Published:** 2014-03-09

**Authors:** Anna Peled, Alexander Pevzner, Hagit Peretz Soroka, Fernando Patolsky

**Affiliations:** 1 School of Chemistry, the Raymond and Beverly Sackler Faculty of Exact Sciences, Tel-Aviv University, Tel Aviv 69978, Israel; 2 The Center for Nanoscience and Nanotechnology, Tel-Aviv University, Tel Aviv 69978, Israel

**Keywords:** Nanowire, Field effect transistors, Chemical stability, Silicon, Dissolution, Biosensors

## Abstract

**Background:**

The detection of biological and chemical species is of key importance to numerous areas of medical and life sciences. Therefore, a great interest exists in developing new, rapid, miniature, biocompatible and highly sensitive sensors, capable to operate under physiological conditions and displaying long-term stabilities (e.g. in-body implantable sensors). Silicon nanostructures, nanowires and nanotubes, have been extensively explored as building blocks for the creation of improved electrical biosensing devices, by virtue of their remarkably high surface-to-volume ratios, and have shown exceptional sensitivity for the real time label-free detection of molecular species adsorbed on their surfaces, down to the sensitivity of single molecules.

Yet, till this date, almost no rigorous studies have been performed on the temporal morphological stability of these nanostructures, and their resulting electrical devices, under physiological conditions (e.g. serum, blood), as well as on the chemical stability of the molecular recognition over-layers covering these structures.

**Results:**

Here, we present systematic time-resolved results on the morphological stability of bare Si nanowire building blocks, as well on the chemical stability of siloxane-based molecular over-layers, under physiological conditions. Furthermore, in order to overcome the observed short-term morpho-chemical instabilities, we present on the chemical passivation of the Si nanostructures by thin metal oxide nanoshells, in the range of 3–10 nm. The thickness of the metal oxide layer influences on the resulting electrical sensitivity of the fabricated FETs (field effect transistors), with an optimum thickness of 3–4 nm.

**Conclusions:**

The core-shell structures display remarkable long-term morphological stability, preventing both, the chemical hydrolytic dissolution of the silicon under-structure and the concomitant loss of the siloxane-based chemical over-layers, for periods of at least several months. Electrical devices constructed from these nanostructures display excellent electrical characteristics and detection sensitivities, with exceptionally high morphological and functional stabilities. These results pave the road for the creation of long-term implantable biosensing devices in general, and nanodevices in particular.

## Introduction

Detection and quantification of chemical and biological species is of key importance to numerous areas of medical and life sciences, from the diagnosis and monitoring of diseases to the discovery of new drugs. The transduction of a signal associated with the specific recognition of the molecules of interest is the key factor to the detection mechanism. Applications of nanotechnology in life sciences have already shown a great potential impact on sensing, diagnostics and drug delivery applications [1-6]. During the last decade, great efforts have been invested in sensing applications based on semiconducting nanostructures [7-11].

Specifically, semiconducting nanowires (NWs), nanotubes and nanoribbons have developed as extraordinarily powerful building blocks, which can be synthesized with a fine control over all their key attributes, such as diameter, length, chemical composition and doping/electrical properties and shape [12-17]. Nanowire-based electronic devices have been recently demonstrated as a powerful and universal sensing platform, demonstrating key advantages such as rapid, direct, highly sensitive multiplexed detection, for a wide-range of biological and chemical species [18,19]. Generally, NW-based electrical devices are transformed into sensors by chemically-linking receptor groups to the surface of the nanostructures, for instance through siloxane-layer formation, rendering them capable of binding to target molecules in a given sample. Upon binding of the analyte species, charges on the captured molecules modulate the nanowire’s surface potential, resulting in changes in the device measured current. These devices, by virtue of their remarkably high surface-to-volume ratios, show an exceptional sensitivity for the detection of molecular species, down to the detection of single molecules [20-24].

However, numerous real-world significant applications (e.g. *in-vivo* implantable sensing devices) strongly require long-term stabilities to be displayed by these nanostructure-based devices under physiological conditions (at 37°C) [24-26]. Additionally, biological studies on the monitoring of long-term intracellular electrical and chemical activity of living cells, as well as in drug delivery applications, may be masked by potential instability of the silicon-based nanoprobes (potentially dissolving into the cells) [26,27]. Yet, the inherent small dimensions of these Si nanostructures may be directly associated with enhanced sensitivity towards their hydrolytic dissolution, thus rendering the resulting electrical devices highly prone to rapid degradability and loss function, as suggested by recent results on the use of silicon-based nanostructures [24].

In this context, two key factors may lead to the incapacity of these devices to act as long-term sensing platforms operating under physiological conditions: (1) the inherent morphological instability of silicon nanostructures though hydrolytic dissolution and (2) the concomitant, or unrelated, chemical instability and dissociation of chemically-anchored recognition layers. The potential morphological degradation, for instance through the slow hydrolytic dissolution of the semiconducting material, of these nanostructures may strongly handicap their future use as building blocks for the creation of long-term sensing devices. Moreover, but not of lesser importance, even the slowest hydrolytic dissolution rates of the underlying semiconducting solid nanomaterial, may jeopardize the integrity of the chemically anchored over-layers (e.g. siloxane-based recognition layers), highly required in the selective detection of molecular and biomolecular species of interest. Although numerous sensing platforms assume that silicon or silicon oxide-based platforms are stable, dissolution of these surfaces even by relatively inert solutions at neutral pH has been reported [28,29]. Thus, several aspects are still required to be investigated in order to determine the applicability of silicon-based nanostructures, as well as of other semiconductors-based structures, on long-term biosensing platforms, operating continuously under physiological conditions.

Here, we present the results of systematic time-resolved studies (using a combination of TEM (transmission electron microscopy), SEM (scanning electron microscopy), XPS (x-ray photoelectron spectroscopy), electrical and sensing measurements) on the morphological stability of bare silicon-based nanostructures, through their chemical dissolution under the given conditions. This was done in parallel to the monitoring and analysis of the chemical stability of siloxane-based over-layers of different nature on the as-grown Si nanowires. Finally, we present on the development of a simple approach for the chemical passivation of Si-based nanostructures by the creation of ultra-thin metal oxide layers, e.g. Al_2_O_3_, in the range of 3–10 nm. Our observations demonstrate that these ultra-stable alumina shell-protected nanostructures remarkably withstand chemical hydrolytic dissolution under physiological conditions, preventing both the dissolution of the underlying silicon nanostructure, as well as the detrimental associated loss of the molecular siloxane-based recognition layers. The improved chemical stability of the molecular over-layers observed may be a result of the remarkably high hydrolytic dissolution-stability of the alumina shell, together with the creation of stronger Si-O-Al, rather than Si-O-Si, bonds created during the binding of silane derivatives to the over-coating alumina shells. By applying this route, SiNW-based electrical sensing devices of exceptionally high morphological and function stabilities are demonstrated, for periods of several months, up to possibly a year.

## Results and discussion

**Figure 1:**
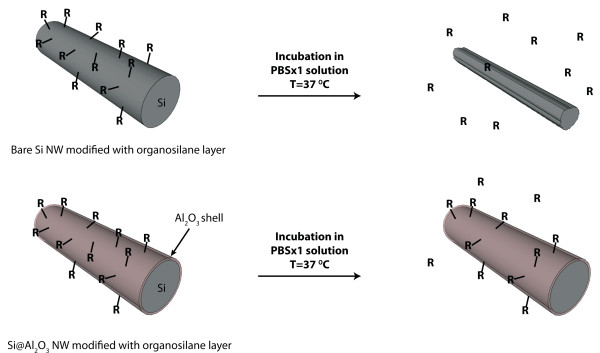
**Nanowires morphological degradation.** Schematic representation of degradation pathways of organosilane-modified Si and Si@Al_2_O_3_ nanowires.

Figure 1 schematically depicts the general characterization approach described in our work. First bare silicon nanowire structures are investigated for their morphological stability under several relevant conditions. Also, the stability of molecular ad-layers, chemically anchored on these surfaces, is studied. Finally, the stability of chemically-passivated silicon nanowire structures, by thin metal oxide layers of control thickness, is investigated and compared to the results observed with bare silicon nanostructures.

Actually, numerous past studies have investigated the hydrolytic stability of silicon oxide and silicon substrates in aqueous solutions. The hydrolytic dissolution of silicon oxide, and of silicon surfaces (though the dissolution of the native oxide layer, followed by subsequent re-oxidation and dissolution cycles) depends on multiple external factors, such as pH, temperature, nature and concentration of electrolytes present in the aqueous solution, as well as the morphology of the silicon oxide/silicon surfaces involved. The hydrolytic dissolution of silicon oxides/silicon in aqueous solutions has been extensively studied [28,30-33], and generally follows the overall reactions:

(1) Si_(s)_ + O_2_ → SiO_2(s)_

(2) SiO_2(s)_ + 2H_2_O_(l)_ → ¼H_4_SiO_4(aq)_

For instance, increasing the concentration of alkaline cations is reported to increase the hydrolytic dissolution rate of silicon oxides [31], as monovalent cations are believed to replace the hydrogen on the surface silanol hydroxyl groups, thus increasing the bond angle and the surface accessibility to water molecules, finally leading to increased rates of hydrolysis [31]. Also, phosphate ions interact with the silicon oxide surface, followed by the nucleophilic attack of these ions, eventually causing the cleavage of the phosphate-silica bonds off the surface. This nucleophilic substitution reaction, leading to dissolution of the solid surfaces, is analogous to that of etching of silicon oxide and silicon surfaces by fluorine ions [34]. In fact, phosphate ions present in physiological solutions have been shown to accelerate the hydrolytic dissolution of silicon and silicon oxide surfaces [30].

**Figure 2:**
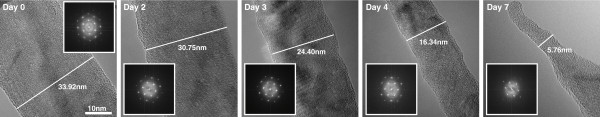
**Unmodified bare SiNWs hydrolytic stability.** HR-TEM images, and corresponding Fast Fourier Transform (FFT) insets, of a typical bare SiNW undergoing degradation, under physiological conditions (PBS × 1 solution, 37°C), within 7 days.

Figure 2 presents TEM (transmission electron microscope) results of the time-resolved investigation of silicon nanowire elements, 20 nm diameter, in a PBS × 1 solution (phosphate buffer saline), at 37°C. This solution mimics physiological conditions, and is currently used in many sensing and biosensing applications. Clearly, the monitoring of single silicon nanowire elements exposed to the mentioned conditions reveals an obvious degradation of the nanowires’ morphological properties, caused by the apparent hydrolytic dissolution of the silicon nanostructures. Notably, the dissolution of the nanowire elements proceeds rapidly, at a rate of ~2.15 nm per day, immediately after exposing the silicon nanowires to the stated solution. Roughening of the nanowires surfaces, along with diameter reduction, is obviously observed almost immediately after nanowires are exposed to this solution under 37°C. This dissolution process leads to non-continuous, segmented, nanowire structures only after 7–8 days of exposure. This rapid dissolution will clearly lead to the failure of electrical devices fabricated using these nanowire building blocks. Notably, continuous exposure of the same nanowire structures in double distilled water and 1 × PBS solutions under ambient conditions (lower temperatures of 20-25°C) lead to considerably lower erosion rates. In water, silicon nanowires do not show a clear decrease in their diameter for periods of several months. Under PBS × 1, nanowires show a slow dissolution rate of 0.1-0.3 nm/day, a rate considerably smaller than observed at 37°C. The higher measured rates of dissolution at 37°C, compared to the same experiment at ambient conditions, is a result of the expected increased rates of hydrolysis at higher temperatures. These results are consistent with most reported studies of silicon nanowire-based electrical devices in sensing and biosensing applications, where the organosilane-modified devices could be continuously used for several days, at least 7–10 days at 37°C, before failure occurred [35].

Functionalization of silicon surfaces with molecules (recognition layer) is critical for the development of bioprobes and biosensors. Among the many surface modification techniques, the formation of a self-assembled monolayers has proved to be very versatile. Routes for surface modification of silicon surfaces frequently make use of silane coupling agents (organosilanes), thus introducing several functional groups such as thiols, epoxides, aldehydes and amines [36]. The resulting organosilane layers simplify the subsequent conjugation of biomolecules, such as oligonucleotides and proteins [36].

Notably, the stability of the formed layers represents the greatest concern in the field of sensing devices, and particularly for long-term implantable sensors. As reported in past and observed in our studies, silicon surfaces are markedly corroded under physiological conditions. Consequently, the biostability of silicon surfaces must be improved if long-term applications (e.g., bio-implantable sensors) are required. To achieve this critical task, the use of organosilane self-assembled layers as a dissolution-protective agents may represent a promising route. Organosilane layers on silicon surfaces may function as passivation layers, by partially suppressing diffusion and surface hydration, preventing their further dissolution [21,27]. The dissolution of the surface will concomitantly lead to the loss of the molecular layers required for sensing applications. In the context of this work we have modified silicon nanowire elements with three different organosilane derivatives, octyl silane (1), APTES aminopropyl triethoxy silane (2) and APDMES aminopropyl dimethyl ethoxy silane (3). Amino-derivatives were shown (through TEM measurements) to partially inhibit the hydrolytic dissolution of the nanowire elements, the extent of which depends on the hydrophilic nature of the silane in use. APTES-modified nanowires showed a dissolution rate of ~1 nm/day in PBS × 1 at 37°C, an approximately 2-fold reduction in the dissolution rate when compared to bare unmodified Si nanowires. The APDMES silane, due to its higher hydrophobicity, effectively reduces the rate of hydrolytic dissolution to ~0.5 nm/day. The highly hydrophobic and dense self-assembled monolayers resulting from the modification of octyl-silane effectively inhibits the further dissolution of the underlying silicon nanostructures, and no obvious measurable dissolution is observed for period of several weeks. The highly hydrophobic monolayers seem to suppress diffusion and surface hydration, and thus inhibit the dissolution of the silicon surfaces. Unfortunately, most sensing applications require the use of functional groups-containing silane derivatives (e.g. amine, thiol), most of which are ineffective in preventing the fast dissolution of the underlying silicon nanowires. The use of mixed monolayers, containing both hydrophobic and hydrophilic silane derivatives, may serve as a potential alternative route to further extend the morphological stability of the silicon nanostructures. Still, applications requiring long-term functional stabilities of nanoelectrical devices, several months to years, will require the development of novel routes of chemically-passivating nanostructures and dramatically improving their hydrolytic stabilities.

With this important goal in mind, we propose the use of core-shell nanowire structures, where the shell material consist on hydrolytically-stable metal oxide layer (e.g. aluminum oxide, zirconium oxide). This layer plays two important tasks: (1) its intrinsic hydrolytic stability prevents the dissolution of the silicon core structures, and (2) serve as a chemically-stable functional platform for the further anchoring of organosilane molecular layers. Replacing the silicon oxide-layer (native oxide layer of silicon nanowires) with the alumina shell is known to effectively suppress ion diffusion and surface hydration, and leads to highly stable layers [37].

**Figure 3:**
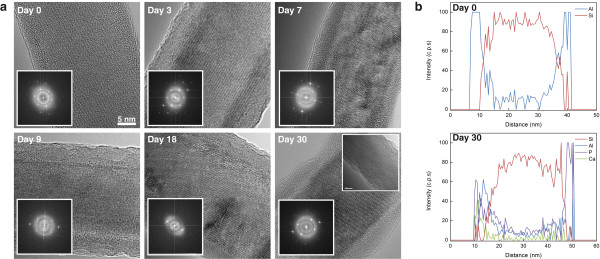
**Alumina-protected SiNWs hydrolytic stability.** Si@Al_2_O_3_ NWs degradation under physiological conditions (PBS × 1 solution, 37°C) within 30 days. **(a)** HR-TEM images and corresponding FFT insets, Bottom TEM inset: after 150 days of incubation, **(b)** EDS line scan analyses of Si@Al_2_O_3_ NWs, before and after 30 days of incubation.

Figure 3a and b shows the time-resolved results of typical silicon-alumina core shell nanowires (~20 nm core diameter and 3 nm alumina shell) continuously exposed to a PBS × 1 solution at 37°C, for a period of 30 days. TEM images clearly display the morphology of the resulting core-shell nanowires, and their time-resolved morphological evolution when exposed to the above-stated conditions. Remarkably, alumina-passivation shells do not show measurable signs of hydrolytic dissolution, with almost constant thickness of ~3 nm along the whole period of incubation. Obviously, no morphological changes occur to the electrically active silicon nanowire elements. These ultra-thin alumina shells can withstand incubation under the given physiological conditions for periods of several months, at least 5 months, before minor morphological signs of erosion are observed. These strikingly stable core-shell nanowire structures may serve as building blocks for the creation of nanoelectrical devices of long-term stabilities.

**Figure 4:**
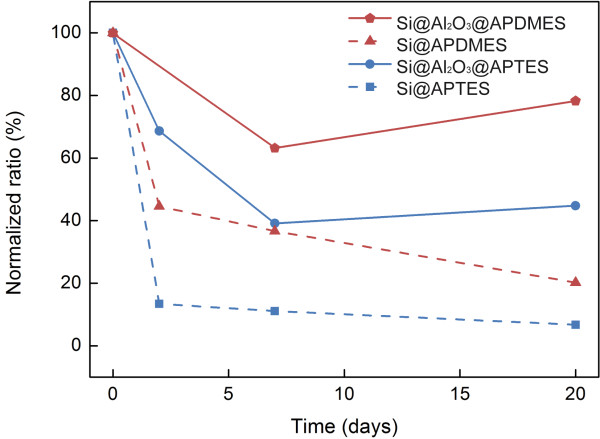
**Stability of molecular siloxane ad-layers.** Time-resolved XPS analyses of chemically-modified native Si (N/Si atomic ratio) and Si@Al_2_O_3_ (N/Al atomic ratio) substrates, incubated in PBS × 1 solution at 37°C.

In order to fully exploit the potential advantages of the hydrolytically-stable silicon-alumina core-shell nanowires, it would be highly desired to demonstrate the capability to produce organo-silane monolayers on alumina surface, along with an enhanced temporal stability of the resulting layers under physiological conditions, as compared to the silicon surfaces. Figure 4 shows the time-resolved XPS results (X-ray photoelectron spectroscopy) of chemically-modified substrates, incubated in PBS × 1 solution at 37°C, for a period of 20 days. Notably, APTES-modified silicon nanowires experience a rapid loss of their chemical over-layers, and being almost entirely de-attached after a short period of only 2–3 days, less than 15% of the initial layer remains. The instability of these layers may be a result of two parallel chemical processes: (1) the highly disordered APTES layers formed on the silicon surfaces cannot effectively isolate the surface from the harsh hydrolytic environment, leading to the fast dissolution of the silicon surfaces and the concomitant loss of the chemically-modified APTES layer[21], and (2) the primary amine group at the end of the APTES molecule is believed to be partially responsible for the instability of the formed layers [21]. It is suspected that the hydrogen bonding between the hydrolyzed APTES molecules and silanol groups (Si–OH) on the Si surface, and among the hydrolyzed APTES monomers, prevent the formation of stable layers. We believe, based on our clear observations on the hydrolytic fast dissolution of silicon nanostructures, that the erosion of the underlying solid surface represents the key reason for the loss of the organosilane layer. A similar rapid degradation tendency is observed for APDMES layers on silicon surfaces. APDMES layers display a higher order and density, along a higher hydrophobicity, and show a slower decay to ca. 20% of the initial layer after only 20 days of incubation under physiological conditions. Both layers, APTES and APDMES, are completely removed after 40 days incubation. Notably, octyl silane-modified surfaces are insensitive to the incubation under these conditions, and no loss of the chemically-modified layer is observed for periods of months. These highly ordered self-assembled dense layers seem to prevent the dissolution of the underlying solid surfaces, through isolation of the surfaces from the aqueous solution, and the concurrent loss of the monolayer (as observed by XPS measurements).

The inherent observed instability of the chemically-anchored organosilane layers, APTES and APDMES, will result in the loss of subsequently immobilized biomolecules, which is very undesirable if such chemically-modified nanostructures are intended for long-term sensing applications under physiological conditions.

Thus, we approached the formation and characterization of organosilane layers on the alumina-passivated silicon surfaces. Figure 4 shows the results of the time-resolved XPS monitoring of APTES and APDMES layers on alumina surfaces. Notably, these layers display a dramatically enhanced stability when compared to that observed for the same layers on silicon surfaces. For instance, APDMES layers on the alumina surfaces experience a slow degradation during the first 5 days, down to ~80% of the initial layer content, after which point they remain remarkably stable for periods of at least several months. This enhanced stability of the molecular over-layers may be a result of the remarkably high hydrolytic dissolution-stability of the alumina layers, together with the creation of stronger Si-O-Al [38,39], rather than Si-O-Si, bonds created during the binding of silane derivatives to the over-coating alumina layers [16,40,41]. Additionally, EDX-TEM measurements reveal the formation of an ultrathin aluminophosphate layer (resulting from the strong interaction between phosphate ion and alumina surface) conformally coating the nanowire’s surface, after incubation of the alumina-coated SiNWs in PBS × 1 solution at 37°C. The formation of this highly stable insoluble layer may further account for the enhanced stability of these core-shell nanowires. [37,39,42] Furthermore, the EDX-TEM measurements reveal the presence of Ca ions, originating from their complexation with aluminaphosphate surface functionalities, after incubation of the alumina-coated nanowires in the PBS solution.

**Figure 5:**
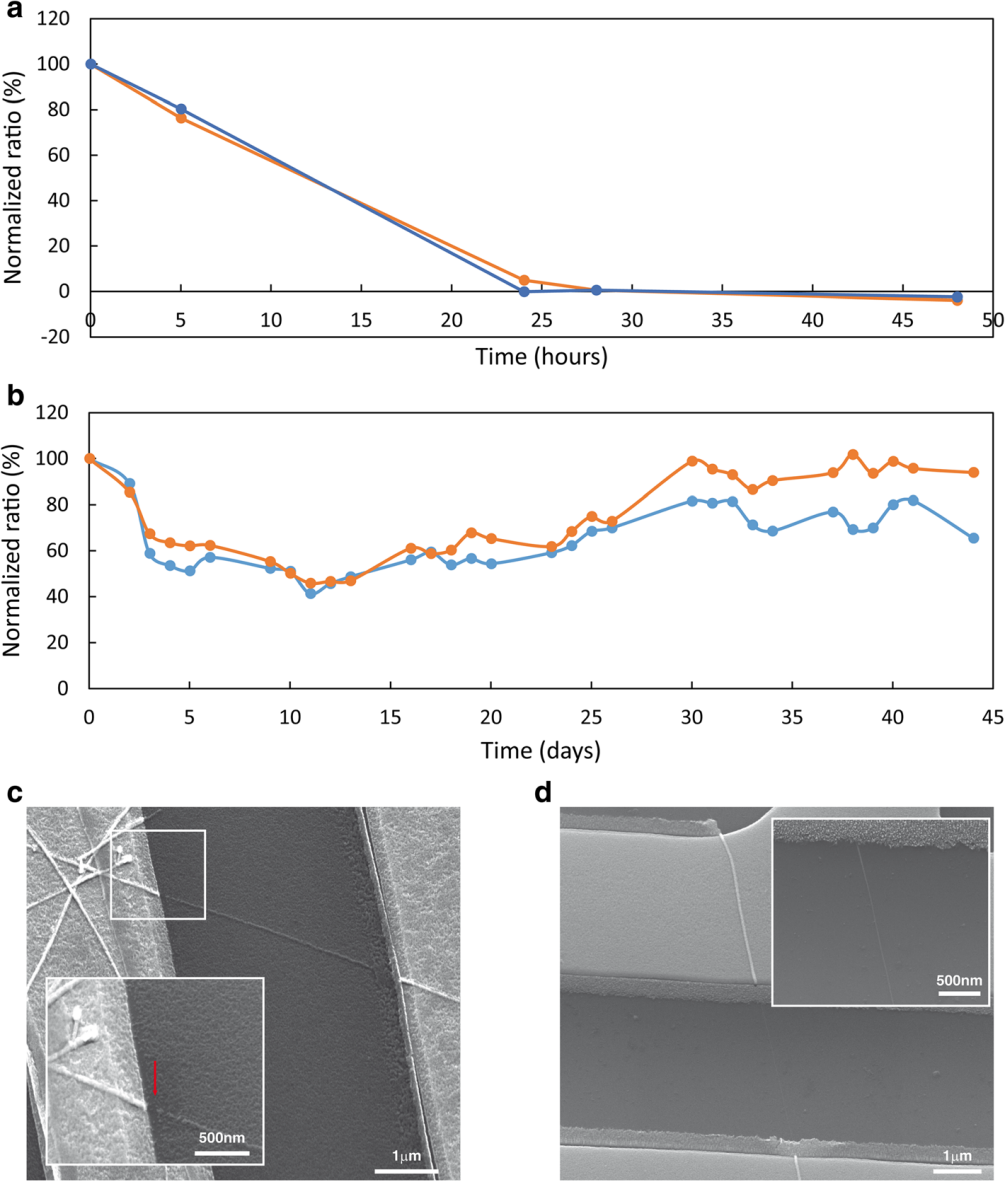
**Time-resolved electrical stability monitoring (normalized transconductance at Vsd = 0.1 V) of two representative FET devices (blue and orange curves) for: uncoated SiNWs (a), and metal oxide protected Si@Al**_**2**_**O**_**3 **_**NWs (b).** SEM images of uncoated SiNW **(c)**, and metal oxide protected Si@Al_2_O_3_ NW devices **(d)**.

The next important step would be the use of these highly stable silicon-alumina core-shell nanowire elements as building blocks for the fabrication of electrical FET (field-effect transistor) devices, and their comparison with the bare silicon-based counterparts. To achieve this goal, electrical nanodevices were fabricated (as described in the Methods Section), and their functional electrical stabilities monitored under continuous operation in PBS × 1 solution at 37°C. Figure 5a shows the time-resolved electrical monitoring of ‘bare’ silicon nanowire-based FET devices under the above stated conditions. Clearly, most devices display a rapid loss of electrical activity after only 2 days of operation. SEM (scanning electron microscopy) imaging of the devices after failure show that the loss of electrical activity is the direct result of hydrolytic dissolution, and loss of continuity, of silicon nanowire bridging units, Figure 5c. Notably, chemically modified nanowire devices, e.g. with APDMES layer, lead to electrical devices of slightly enhanced stabilites, functional up to 7 days under these conditions. Clearly, electrical devices based on bare-silicon nanowires cannot serve as candidates for the creation of future long-term stable nanodevices.

Alternatively, as shown in Figure 5b and d, the use of alumina-coated silicon nanowires building blocks (20 diameter silicon cores, 3 nm alumina coating), leads to the creation of electrical nanodevices extraordinarily more stable than the bare-silicon nanowire counterparts. Continuous electrical monitoring of alumina-coated SiNWs devices shows that the resulting devices are electrically stable for periods of several months, with functional degradation of ca. 50% after 5 months of continuous operation under physiological conditions. SEM observations of these devices clearly show that the alumina-coated nanowires are stable throughout the whole monitoring period, nanowires staying as continuous bridging elements.

**Figure 6:**
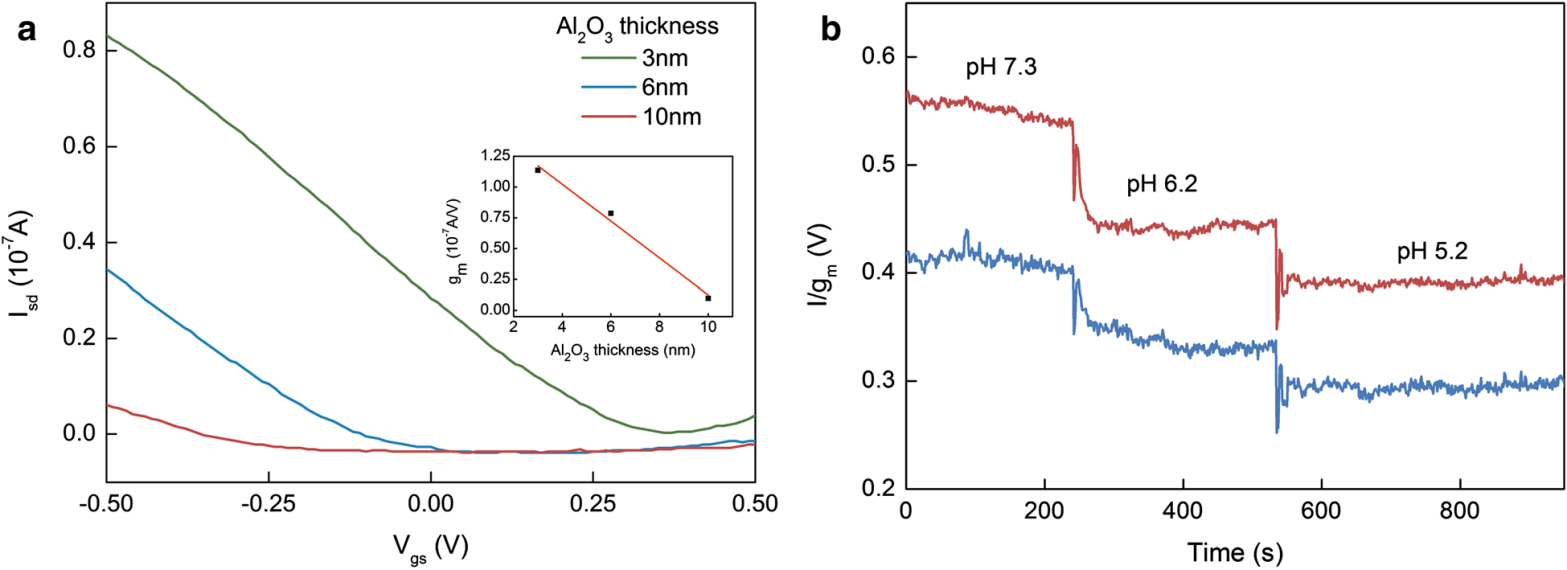
**Stability versus sensitivity of FET devices. (a)** IV curves of two representative SiNW FET devices coated with various thickness of alumina protecting layers (blue and red curves), and their corresponding water-gate transconductance values (inset). **(b)** Real time sensing of pH with two representative 3 nm alumina-coated SiNW FET devices (blue and red curves), at constant *V*_g_ = −0.1 V.

Additionally, the intrinsic sensitivity of nanowire-based devices is one of the key advantages when applying these devices in sensing applications. Undoubtedly, the use of alumina passivation layers of higher thickness may lead to electrical devices of longer stabilities, but may in parallel bring to the electrical isolation of the resulting nanowire devices from the sensing environment. This may cause a dramatic decrease in the sensitivity of the resulting electrical FETs, and may impede their further use as sensing devices. Thus, a trade-off between the optimum thickness of alumina layer, in order to achieve the best long-term stability, and the highest electrical sensitivity achievable must be taken into account. In this context, we have tested the electrical response of different nanowire devices, in terms of their water-gate transconductances, before and after the deposition of alumina layers of increasing thickness, from 3 to 10 nm, Figure 6a. Obviously, increasing the thickness of the alumina coating leads to a dramatic decrease in the measured transconductance values and device electrical sensitivity. Notably, devices consisting of 10 nm alumina layer, and higher, are almost insensitive to water-gate modulation, being buried under the thick metal-oxide layer that electrically isolates the silicon nanowire sensing channels from the surrounding aqueous environment. These devices are poorly sensitive to pH changes of the measuring electrolyte. Chemical etching of the alumina layer, in NaOH solution, renders exposed bare silicon nanowire devices that display high transconductances and pH sensitivities, similar to the devices before deposition of alumina layer Figure 6b. These results demonstrate the importance in controlling the thickness of the metal oxide coating layer in achieving the best hydrolytic long-term stabilities, while retaining the highest possible electrical sensitivities.

## Conclusions

This work presents several key observations regarding the morphological hydrolytic stability of silicon nanostructures under physiological conditions, as follows:

(1) Bare silicon nanowire structures display a rapid hydrolytic dissolution when exposed to PBS × 1 solution at 37°C. Nanowire elements undergo a rapid erosion process at a dissolution rate of ca. 2 nm/day, leading to the complete dissolution of the silicon structures after approximately 8–10 days. (2) Chemical modification of silicon nanowire elements, with different organosilane molecular layers, passivates the silicon surfaces and moderately inhibits the hydrolytic dissolution, a rate which depends on the nature of the organosilane molecular layer. (3) Bare silicon nanowires do not undergo considerable erosion in water and in PBS × 1 solutions under ambient temperatures (20-25°C) for long period of time, thus temperature pays an important role in the measured rapid hydrolytic dissolution rates. (4) Organosilane layers anchored to silicon nanowire structures display limited stabilities, almost completely detaching from the solid surfaces after 2–3 days of incubation in PBS × 1 solution at 37°C. These observations are attributed to both, the fast erosion of the underlying solid substrates and the limited instability of the organosilane resulting layers. (5) Electrical FET devices fabricated from silicon nanowire elements present a rapid degradation in the electrical functionality, in PBS × 1 solution at 37°C, showing complete failure after only 3 days of continuous operation. This situation is partially attenuated by chemically-modifying the silicon nanowire elements with organosilane layers. Failure is clearly attributed to the hydrolytic erosion of the nanowire bridging units under the given conditions. (6) These results demonstrate the inability to use silicon nanowires as building blocks for the fabrication of future long-term stable sensing devices.

In order to overcome this handicapping limitations, we developed a novel approach for the chemical passivation of silicon nanostructures, based on the deposition of ultra-thin shell of metal oxides, e.g. aluminum oxide, resulting in the formation of hydrolytically-stable core-shell structures. Silicon nanowire elements covered with a thin 3 nm alumina coating layer display dramatically enhanced hydrolytic stabilities, resulting in core-shell nanowire structures stable, against hydrolytic dissolution, for periods of several months long, at least 5 months. These enhanced stabilities are attributed to the inherent suppression of diffusion and surface hydration of alumina surfaces, in comparison to silicon oxide surfaces, preventing their further dissolution. Notably, organosilane layers formed on alumina-covered silicon surfaces display dramatically enhanced stabilities, and largely remain anchored to the alumina surfaces for periods of several months. Furthermore, the maximum thickness of the alumina layer is strongly dictated by the required electrical sensitivity of the resulting nanodevices. Alumina layers of ~10 nm and higher result in electrically-insensitive devices, not suitable for sensing applications where high sensitivities are required. Thus, a trade-off between the maximum long-term stability of the nanowire-devices and their resulting sensitivity is required, when deciding on the thickness of the deposited metal oxide layer.

Generally, our measurements demonstrate the capability to fabricate highly-sensitive silicon nanowire-based electrical FET devices that display strikingly high long-term hydrolytic stabilities (several months to possibly a year), suitable for future implantable sensor applications.

## Materials and methods

### Synthesis of SiNWs

p-type silicon nanowires (SiNWs) were prepared by chemical vapor deposition (CVD) as was reported previously.[43] Briefly, 20 nm gold nanoparticles (Ted Pella), which served as catalyst sites for the VLS-CVD growth of Si nanowires, were initially deposited on Si (100) growth substrates. To promote the adhesion of the gold nanoparticles to the silicon substrate, a poly-L-lysine solution (Ted Pella) was applied to the bare silicon wafer, as an electrostatic binding agent. The nanoparticle-decorated wafer was then placed in a horizontal tube furnace for the growth of the SiNWs. Silane and diborane were used as reactants during the growth to provide boron as a p-type dopant with a B:Si ratio of 1:4000.

### Alumina passivation via Atomic Layer Deposition (ALD)

Alumina coatings were deposited using Savannah 200 ALD system (Cambridge NanoTech Inc). Layers were grown by repeated pulses of trimethylaluminum (TMA) and water at 80°C (cycle sequence: TMA 0.015 s, wait 5 s, H_2_O 0.015 s, wait 5 s) using Nitrogen 99.9999 as carrier gas at 20 sccm flow rate. Each cycle produces ~1.1 Å of Al_2_O_3_ at 80°C. It should be noted, that conditions for ALD deposition were same for all studies performed in this work.

### Chemical stability of SiNWs

Bare SiNWs and SiNWs@Al_2_O_3_ were incubated in PBSx1 solution at 37°C for certain time and rinsed with double-distillated water (DDW) before TEM studies. Next, SiNWs were stripped off from the growth substrate by sonication in isopropanol and then deposited on lacey carbon copper grid (Ted Pella, Inc.) for TEM studies. For single NWs experiment, lacey carbon gold grid (Ted Pella, Inc.) was incubated for certain time in PBS × 1 solution at 37°C, rinsed with DDW, studied by TEM, and incubated again, under the same conditions.

TEM studies for checking stability of bare SiNWs as well as SiNWs@Al_2_O_3_ were performed on Philips Tecnai F20 FEG microscope. Energy Dispersive X-ray Spectroscopy (EDS) line scan analyses were performed on Titan 80–300 FEG-S/TEM (FEI) microscope.

### Chemical stability of organosilane layers

Native Si and Si@Al_2_O_3_ substrates were modified using two types of organosilanes: (3-aminopropyl) triethoxysilane (APTES,) and (3-aminopropyl) dimethylethoxysilane (APDMES). Organosilanes were purchased from Gelest, Inc.

#### Modification with APTES layer

First, 200 μL of APTES were added to 20 mL of EtOH/H_2_O (95%/5%) solution. Resulted solution was allowed to stand for 20 minutes before filtering through a cutoff syringe filter (cellulose, 0.2 μm, Whatman). Then, substrates were immersed in filtered solution for 30 min at room temperature. Finally, modified substrates were thoroughly rinsed with EtOH/H_2_O (95%/5%) solution, rinsed with EtOH, dried under a stream of nitrogen and baked at 110°C for 10 min.

#### Modification with APDMES layer

First, APDMES organosilane was dropped on the native Si or Si@Al_2_O_3_ substrate to cover it completely. Then substrates were baked at 60°C for 60 min. Finally, modified substrates were thoroughly rinsed with IPA, dried under a stream of nitrogen and baked at 110°C for 10 min.

### X-ray Photoelectron Spectroscopy (XPS) measurements

XPS measurements were performed in UHV (2.5 × 10–10 torr base pressure) using 5600 Multi-Technique System (PHI, USA). The samples were irradiated with an Al K_α_ monochromated source (1486.6 eV) and the outcome electrons were analyzed by a Spherical Capacitor Analyzer using the slit aperture of 0.8 mm. Neutralizer was not used. The samples were analyzed at the surface only. Measurements were performed at 23° take-off angles. Modified substrates were incubated in PBS × 1 solution at 37°C for certain period, rinsed with DDW, and studied by XPS.

### SiNW-FET device fabrication

SiNW-FET device was fabricated by standard photolithography procedures as was reported previously [41]. Briefly, outer pads and gates were defined using multilayer photoresist (~500 nm LOR-5A and ~500 nm S-1805; MicroChem Corp.) using an outer mask, followed by thermal evaporation of Cr/Au (5/60 nm) respectively, and lift off using PG remover. Next, source and drain electrodes were defined using the same photoresists and were metallized by e-beam evaporation of Ti/Pd/Ti (5/60/5 nm) respectively. Next, device was coated with an insulating layer of silicon nitride (~50 nm) deposited by Inductively Coupled Plasma Enhanced Chemical Vapor Deposition (ICP-PECVD) followed by lift off in PG remover.

### Alumina-passivated SiNW FET devices

Outer pads and gates of as prepared SiNW-FET were protected with ~ 1.6 μm of AZ-5214 (AZ Electronic Materials) image-reversal photoresist, using the outer mask by photolithography. Then, 3–10 nm alumina layer was deposited by ALD, followed by lift off in PG remover.

### Electrical sensing and temperature-control setup

All studies were carried out under physiological conditions (PBSx1 solution at 37°C). FET device was continuously heated, while temperature control was carried out by using the PWM outputs to drive a heater cartridge using PID control loop, while the temperature is sensed by a thermistor unit and read by ADC [3]. The sensor device was integrated with a custom-made PDMS microfluidic channel and wire bonded to the outside pads for the electrical measurements. The electrical stability of the devices was measured by application of AC bias (70-300Hz, 100 mV) by means of a lock-in amplifier (Stanford Research System model SR830 DSP). The drain current was amplified with a variable-gain amplifier (model 99539 Amplifier System) and filtered by the lock-in amplifier with a time-constant setting of 300 ms. The output data were recorded by using a multichannel I/O adaptor panel (BNC-2090, National Instrument). Stability studies were carried out by monitoring the IV (−0.5 V to 0.5 V) of the devices over time, while PBS × 1 working solution was delivered to the sensing device by the microfluidic system using a syringe pump at a flow rate of 2 μl/min.

## Competing interests

The authors declare that they have no competing interests.

## Authors’ contributions

AP (Anna Peled) carried out experiments and analyzed data. AP (Alexander Pevzner) carried out experiments and analyzed data as well assisted with manuscript preparation. HPS assisted with electrical experiments. FP conceived of the study, analyzed data and wrote the manuscript. All authors read and approved the final manuscript.
